# Outcome of older patients with cancer requiring ICU admission

**DOI:** 10.1186/cc12408

**Published:** 2013-03-19

**Authors:** L Hajjar, M Franca Cavalcante, F Galas, JT Fukushima, MR Sundin, F Bergamin, C Park, H Palomba, DM Goto, A Tavares, M Santos, A Freitas, P Tonini, L Cavichio, JP Almeida, M Bazan

**Affiliations:** 1 Heart Institute, São Paulo, Brazil; 2 Cancer Institute of University of São Paulo, Brazil

## Introduction

Because the prognosis of older patients with cancer may be poor compared with younger patients, it remains controversial whether they benefit from ICU treatment. The objective of this study was to identify factors associated with hospital mortality in older patients with cancer requiring the ICU.

## Methods

A retrospective study was conducted in consecutive medical and surgical older patients with cancer admitted to a university hospital ICU in São Paulo, between 2007 and 2012. Univariate and multivariate analysis were performed to identify associated and independent factors related to hospital mortality.

## Results

From 8,976 patients with cancer requiring ICU at the period, 600 patients were 80 years old or higher. Most patients were male (53%), had solid neoplasm (86%), were from medical admission (54%) and 37% had metastatic disease. The mean age was 84 years (± 3). The ICU and hospital mortality rates were 30% and 44%, respectively. In the univariate analysis, variables associated with hospital mortality were diagnosis of lung cancer, medical admission, active neoplasm, vasopressor need at 24 hours of ICU, acute renal failure at admission, mechanical ventilation need at admission to and at 24 hours of ICU and a higher admission arterial lactate. By multivariate analysis, risk factors of hospital mortality were diagnosis of lung cancer (OR = 2.32; 95% CI, 1.08 to 3.29, *P *0.001), medical admission (OR = 6.473; 95% CI, 2.76 to 15.15, *P *0.001), acute renal failure at admission (OR = 3.09; 95% CI, 1.49 to 9.21, *P *0.001), mechanical ventilation at 24 hours of ICU (OR = 5.68; 95% CI, 2.87 to 7.26, *P *0.001) and lactate levels at admission (OR = 1.02; 95% CI, 1.006 to 1.051, *P *0.001).

## Conclusion

Hospital survival in older patients with cancer requiring ICU admission is acceptable. Our results provide evidence that ICU management may be appropriate in older patients with cancer and appoint risk factors for mortality, helping to better triage cancer patients who will benefit from ICU care.

